# Long-term outcomes of anthroposophic treatment for chronic disease: a four-year follow-up analysis of 1510 patients from a prospective observational study in routine outpatient settings

**DOI:** 10.1186/1756-0500-6-269

**Published:** 2013-07-13

**Authors:** Harald Johan Hamre, Helmut Kiene, Anja Glockmann, Renatus Ziegler, Gunver Sophia Kienle

**Affiliations:** 1 Institute for Applied Epistemology and Medical Methodology at the University of Witten-Herdecke, Zechenweg 6, D-79111, Freiburg, Germany; 2 Society for Cancer Research, Kirschweg 9, CH-4144, Arlesheim, Switzerland

## Abstract

**Background:**

Anthroposophic treatment includes special artistic and physical therapies and special medications. We here report an update to a previously published study of anthroposophic treatment for chronic diseases, including more patients and a longer follow up. The Anthroposophic Medicine Outcomes Study (AMOS) was a prospective observational cohort study of anthroposophic treatment for chronic indications in routine outpatient settings in Germany. Anthroposophic treatment was associated with improvements of symptoms and quality of life. Previous follow-up-analyses have been performed after 24 months or, in subgroups of patients enrolled in the period 1999-2001, after 48 months. We conducted a 48-month follow-up analysis of all patients enrolled in AMOS in the period 1999-2005.

**Methods:**

1,510 outpatients aged 1-75 years, starting anthroposophic treatment for chronic conditions in routine German outpatient settings, participated in a prospective cohort study. Main outcomes were Symptom Score (primary outcome, mean symptom severity on numerical rating scales), SF-36 Physical and Mental Component scores in adults, and disease-specific outcomes in the six most common diagnosis groups: asthma, anxiety disorders and migraine (numerical rating scales), depression (Center for Epidemiological Studies Depression Scale), attention deficit hyperactivity symptoms (FBB-HKS Total score), and low back pain (Hanover Functional Ability Questionnaire, Low Back Pain Rating Scale).

**Results:**

Median disease duration at baseline was 3.5 years. From baseline to 48-month follow-up all ten outcomes improved significantly (p < 0.001 for all pre-post comparisons). Standardised Response Mean effect sizes were large (range 0.84-1.24 standard deviations) for seven comparisons, medium for two comparisons (SF-36 Mental Component: 0.60, Low Back Pain Rating Scale: 0.55), and small for one comparison (SF-36 Physical Component: 0.39). Symptom Score improved significantly with large effect sizes in adults and children, and in the four main anthroposophic therapy modality groups (art therapy, eurythmy therapy, rhythmical massage therapy, medical therapy).

**Conclusions:**

This 48-month follow-up analysis confirmed previous analyses from the AMOS study. Outpatients receiving anthroposophic treatment for chronic indications had sustained, clinically relevant improvements of symptoms and quality of life.

## Background

We here report an update to a previously published study of anthroposophic treatment for chronic diseases [1], including more patients and a longer follow up.

Chronic diseases are the most common cause of disease burden worldwide and are rarely completely cured [2]. Many patients with chronic disease use complementary therapies [3], often provided by their physicians.

Anthroposophic medicine (AM), founded by Rudolf Steiner and Ita Wegman in the 1920s [4], is a complementary therapy system. AM acknowledges a spiritual-existential dimension in man, which is assumed to interact with psychological and somatic levels in health and disease. AM therapy for chronic disease aims to counteract constitutional vulnerability, stimulate salutogenetic self-healing capacities, and strengthen patient autonomy [5-7]. This is sought to be achieved by counselling [6]; by non-verbal artistic therapies using painting or clay [8-10], music [11] or speech exercises [12]; by eurythmy movement exercises [13,14]; by special physical therapies [15,16]; and by special AM medications [17,18].

AM is provided by physicians (counselling, medications) in collaboration with non-medical practitioners (e.g. eurythmy, art therapy, physical therapy) in hospitals and outpatient settings. AM treatment is used alone or in combination with conventional treatment as needed. In Europe 24 hospitals (including 14 with accident & emergency services) offer AM therapy, and an estimated 17.000 physicians in outpatient settings prescribe AM medications [19].

Since AM therapy aims for sustained improvement [6,7], long-term clinical outcomes are particularly important in the evaluation of AM therapy. An opportunity to study such long-term outcomes was given by the Anthroposophic Medicine Outcomes Study (AMOS). AMOS was a prospective observational cohort study of AM therapy for chronic indications in routine outpatient settings in Germany [1]. The study was initiated by a health insurance company in conjunction with a health benefit program. AM treatment was associated with improvements of symptoms and quality of life. Previous follow-up-analyses have been performed after 24 months or, in subgroups of patients enrolled in the period 1999-2001, after 48 months [1,20-30]. We here present a 48-month follow-up analysis of all patients enrolled in AMOS in the period 1999-2005.

## Methods

### Study design and research questions

This is the final follow-up analysis of a prospective observational cohort study of AM therapy for chronic diseases in routine outpatient settings (AMOS) [1]. Compared to previous outcome analyses of the AMOS study, the present analysis was an extension regarding: 

•recruitment period: 1999-2005 vs. 1999-2001 in previous analyses [1,20-24]

•last follow-up: 48 months vs. 24 months in previous analyses [1,25-30]

The research questions concerned the following items:

A. Disease symptoms and quality of life at 48- month follow-up (main research question)

B. Patient satisfaction after 6 and 12 months

C. Safety in months 0-24

D. Continuity of the physician-patient relationship at 48-month follow-up.

For items A-C, the research question was: Can findings from previous analyses that were restricted to patient subgroups (items A-C) or earlier follow-ups (item A) be confirmed in the present full sample and at 48 months follow-up, respectively?

For item D, previously not analysed, the research question was: Which proportion of the patients are, after 48 months, still being treated by the same physician who had enrolled them into the study?

### Setting, participants, and therapy

All physicians certified by the Physicians’ Association for Anthroposophical Medicine in Germany and working in an office-based practice or outpatient clinic in Germany were invited to participate in the AMOS study. The participating physicians were asked to recruit consecutive outpatients starting AM therapy under routine clinical conditions. Patients enrolled from 1 January 1999 to 31 December 2005 were included in the present analysis if they fulfilled the eligibility criteria (patients enrolled before 1 January 1999 had no follow-up documentation beyond 12 months). Inclusion criteria were:

1. Outpatients aged 1-75 years.

2. Starting AM therapy for any indication (main diagnosis)

2a: AM-related consultation of at least 30 minutes followed by new prescription of AM medication, or

2b: referral to AM treatment by non-medical therapist: art therapy, eurythmy therapy or rhythmical massage therapy.

3. Duration of main diagnosis of at least 30 days at study enrolment.

Patients were excluded if they had previously received the AM therapy in question (see inclusion criteria no. 2) for their main diagnosis. AM therapy was evaluated as a whole system [31] with subgroup analyses of adults and children and in previously published diagnosis groups [24,26-30] and therapy modality groups [20-23]. In subgroup analyses of patients according to AM therapy modality, patients fulfilling inclusion criteria 2a as well as 2b were analysed in group 2b.

### Clinical outcomes

Clinical outcomes were documented after 0, 3, 6, 12, 18, 24, and 48 months. In this analysis clinical outcomes were assessed at 48-month follow-up.

#### *Primary outcome*

•Primary outcome was Symptom Score, a compound measure of the symptoms for which the patients had sought medical attention. At baseline, the patients (caregivers in children) documented one to six symptoms, ranked in order of decreasing importance, and assessed the intensity of each symptom on a numerical rating scale [32] from 0 (“not present”) to 10 (“worst possible”). At each follow-up, the patients documented the intensity of the same symptoms which they had documented at baseline. Symptom Score was the average severity of all documented symptoms per patient at each documentation point. This symptom rating was used as primary outcome measure for the present analysis because it was the only clinical outcome documented in all patients at 48-month follow-up.

#### *Secondary outcomes*

•First ranked symptom at baseline (see above) on a numerical rating scale 0-10 [32]

•Generic quality of life: SF-36 Physical and Mental Component summary measures and the eight SF-36 scales (0-100) [33] for adults aged 17-75 years (quality of life was not documented in children at 48-month follow-up)

•Depressive symptoms: Center for Epidemiological Studies Depression Scale, German version (CES-D, 0-60) [34,35] for adults enrolled after 1 October 1999 (depressive symptoms were not documented in children, and CES-D was not documented at 48-month follow-up in patients enrolled before 1 October 1999)

•Disease-specific outcomes for the most frequent diagnosis groups: Anxiety Disorders, Asthma, Attention Deficit Hyperactivity Symptoms, Depression, Low Back Pain, and Migraine

#### *Other outcomes*

•Use of AM art, eurythmy and rhythmical massage therapy in months 0-24 was documented by the therapists in a therapy diary.

•Therapy outcome rating and satisfaction with therapy were documented by the patients on numerical rating scales (0-10) after 6 and 12 months.

•The continuity of the physician-patient relationship was documented after 6, 12, 18, 24, and 48 months.

•Suspected adverse reactions to medications or therapies were documented by the patients after 6, 12, 18, and 24 months, and by the physicians after 6 months (for patients enrolled before 1 April 2001 also after 3, 9, and 12 months). The documentation included suspected cause, intensity (mild/moderate/severe = no impairment/some impairment/complete impairment of normal daily activities), and therapy discontinuation because of adverse reactions.

•Serious adverse events (death, life-threatening condition, acute in-patient hospitalization, new disease or accident causing permanent disability, congenital anomaly, new malignancy) were documented by the physicians throughout the study months 0-24.

### Data collection

All data were documented with questionnaires returned in sealed envelopes to the study office. The physicians documented eligibility criteria; all other items were documented by the patients or caregivers, unless otherwise stated. The patient responses were not made available to the physicians. The physicians were compensated 40 Euro (after March 2001: 60 Euro) per included and fully documented patient, while the patients received no compensation.

The data were entered twice by two different persons into Microsoft® Access 97. The two datasets were compared and discrepancies resolved by checking with the original data.

### Quality assurance, adherence to regulations

The study was approved by the Ethics Committee of the Faculty of Medicine Charité, Humboldt University, Berlin, Germany, and was conducted according to the Declaration of Helsinki and largely following the ICH Guideline for Good Clinical Practice E6. Written informed consent was obtained from all patients before enrolment.

### Data analysis

The data analysis was performed on all patients fulfilling the eligibility criteria, using PASW® Statistics 18.0 (SPSS Inc., Chicago, Ill, USA) and StatXact® 9.0.0 (Cytel Software Corporation, Cambridge, MA, USA). Diagnoses were coded according to the International Classification of Diseases, Tenth Revision (ICD-10).

For bivariate analyses of continuous data with approximately normal distribution the two-tailed *t*-test was used; for independent samples with skewed data the Mann–Whitney *U*-test was used. For binominal data the two-tailed McNemar test and Fisher’s exact test were used. Significance criteria were p < 0.05. Since this was a descriptive study, no adjustment for multiple comparisons was performed [36].

Pre-post effect sizes were calculated as Standardised Response Mean (= mean change score divided by the standard deviation of the change score) and classified as minimal (< 0.20), small (0.20-0.49), medium (0.50-0.79), and large (≥ 0.80) [37,38]. Clinical relevance criteria for pre-post changes were Standardised Response Mean ≥ 0.50 [39].

For analysis of clinical outcomes, missing values were replaced with the last value carried forward. Accordingly, each clinical outcome and subgroup analysis comprised all patients in the respective group with available baseline scores (in total 98.7%, n = 17,994 of 18,223 scores for 26 outcome analyses). For other analyses, missing data were not replaced.

All suspected adverse reactions were classified as reported adverse reactions and subject to descriptive analysis. The frequency of reported reactions from AM medications in this analysis (patients enrolled January 1999 to December 2005) were compared to the frequency of confirmed reactions in a previously published detailed safety analysis from AMOS (patients enrolled January 1999 to March 2001) [40]. Serious adverse events were assessed with regard to a possible causal relationship to ongoing medication and non-medication therapy by the study physicians and the first author.

## Results

### Participating physicians and therapists

A total of 151 physicians enrolled patients. These physicians did not differ significantly from eligible physicians without study patients (n = 167) regarding gender (proportion of male physicians: 57.0% vs. 64.7%, p = 0.134) and setting (proportion of primary care physicians: 85.4% vs. 82.1%, p = 0.876). Significant differences were found regarding the number of years in practice (mean ± standard deviation 18.0 ± 7.4 years vs. 20.3 ± 9.5 years in physicians with and without study patients, respectively, p = 0.011, mean difference 2.3 years, 95% confidence interval [95%-CI] 0.5-4.0 years) and age (46.8 ± 7.0 years vs. 49.5 ± 8.5 years, p = 0.003, mean difference 2.6 years, 95%-CI 0.9-4.4 years).

The patients who had been referred to AM art, eurythmy or rhythmical massage therapy were treated by 275 different therapists. Comparing these therapists to eligible therapists without study patients (n = 911), no significant differences were found regarding age (mean 49.2 ± 8.1 years vs. 50.6 ± 9.8 years, p = 0.068), gender (81.5% vs. 80.7% women, p = 0.861) or the number of years since therapy school graduation (12.1 ± 7.1 vs. 13.5 ± 9.2 years, p = 0.225).

### Patient recruitment and follow-up

From 1 January 1999 to 31 December 2005, a total of 1,678 patients aged 1-75 years were assessed for eligibility. Of these patients, 1,510 fulfilled all eligibility criteria and were included in the present analysis. Of the 168 patients who were not included, 32 did not fulfil the eligibility criteria for the analysis (disease duration < 30 days: n = 32) and 136 patients were potentially eligible but were not included in the AMOS study for the following reasons: patients’ baseline questionnaire missing (n = 57), patients’ and physicians’ baseline questionnaire dated > 30 days apart (n = 49), previous or ongoing use of AM therapy (n = 19), no informed consent (n = 8), other reasons (n = 3). The included patients (n = 1,510) and the patients not included but potentially eligible (n = 136) did not differ significantly regarding age, gender, diagnosis, disease duration or baseline Symptom Score.

A total of 71.7% (n = 1,083/1,510) of patients were enrolled by general practitioners, 12.2% by paediatricians, 6.4% by internists, 6.1% by gynaecologists, and 3.6% by other specialists. The physicians’ settings were primary care practices (82.1% of evaluable patients, n = 1,212/1,477), referral practices (11.2%), and outpatient clinics (6.6%). Each physician enrolled 1-4 patients (51.0%, n = 77/151 physicians), 5-9 patients (21.9%, n = 33), 10-19 patients (15.2%, n = 23) or ≥ 20 patients (11.9%, n = 18), with a median of 4.0 patients enrolled per physician (range 1-55 patients, interquartile range [IQR] 2.0-11.0 patients, average 10.0 patients).

The last patient follow-up ensued on 16 May 2010. A total of 96.0% (n = 1,450/1,510) of patients returned at least one follow-up questionnaire. The patients were administered a total of 9,060 follow-up questionnaires, out of which 7,114 (78.5%) were returned. Follow-up rates were 94.5% (n = 1,377/1,510), 91.2%, 86.7%, 82.9%, 76.6%, 73.2%, and 60.5% after 3, 6, 12, 18, 24, and 48 months, respectively. Respondents (= patients who did return the follow-up questionnaire, n = 914) and non-respondents (n = 596) of the 48-month follow-up did not differ significantly regarding age, gender, diagnosis or disease duration. Baseline Symptom Score was 6.0 ±1.8 points in respondents and 6.3 ±1.7 points in non-respondents (p < 0.001, mean difference 0.3 points, 95%-CI 0.2-0.5 points). For non-respondents of the 48-month follow-up, the last available questionnaire was returned after average 15.7 months. In respondents of the 48-month follow-up Symptom Score was (interpolated average) 3.40 points after 15.7 months and 2.48 points after 48 months.

### Baseline characteristics

The patients were recruited from 15 of 16 German federal states. Age groups were 1-19 years: 29.8% (n = 450/1,150), 20-39 years: 25.4%, 40-59 years: 35.2%, and 60-75 years: 9.6% with a median age of 37.0 years (IQR 12.3-47.1 years, mean 33.8 ± 19.4 years). A total of 69.8% (n = 1,054/1,510) of all patients and 81.5% (n = 975/1,074) of adults aged 17-75 were women.

Compared with the German population, adult patients had higher educational and occupational levels and were less frequently regular smokers, daily alcohol consumers, overweight, and unemployed, whereas more patients engaged in sports than in the population. Socio-demographic status was similar to the population regarding income, severe disability status, and the proportion living alone, and less favourable for work disability pension and sick-leave (Table 1). Socio-demographic characteristics of the children have been presented elsewhere [25].

**Table 1:** Socio-demographic characteristics of adult patients (age 17-75 years, n = 1074)

**Item**	**Subgroup**	**Patients**		**German population**	
		**N**	**%**	**%**	**Reference**
Education [41]					[42]
-Low (grade 1)		184	17.1%	43%	[43]
-Intermediate (grade 2)		530	49.3%	43%	
-High (grade 3)		360	33.5%	14%	
Wage earners		39/1074	3.6%	18%	[42]
Unemployed during last 12 months	Economically active patients	37/618	6.0%	10%	[42]
Living alone		208/1069	19.5%	21%	[42]
Net family income < 900 € per month		160/871	18.4%	16%	[42]
Alcohol use daily (patients) vs almost daily (Germany)	Male	12/198	6.1%	28%	[44]
	Female	19/875	2.2%	11%	
Regular smoking	Male	26/199	13.1%	37%	[45]
	Female	92/873	10.5%	28%	
Sports activity ≥ 1 hour weekly	Age 25-69	484/887	54.6%	39%	[46]
Body mass index < 18.5 (low)	Male	7/196	3.6%	1%	[42]
	Female	61/863	7.1%	4%	
Body mass index ≥ 25 (overweight)	Male	64/196	32.7%	56%	[42]
	Female	219/863	25.4%	39%	
Permanent work disability pension		204/1072	19.0%	3%	[47]
Severe disability status		116/1072	10.8%	12%	[48]
Sick leave days in the last 12 months: mean (standard deviation)	Economically active patients	32.6 (67.3)		17.0	[49]

Most frequent main diagnoses, classified by the ICD-10 diagnosis chapters were F00-F99 Mental and behavioural disorders (35.2%, n = 532/1,510), M00-M99 Diseases of the musculoskeletal system and connective tissue (15.4%), J00-J99 Diseases of the respiratory system (9.9%), and G00-G99 Diseases of the nervous system (7.2%). The most frequent diagnosis groups are listed in Table 2. Disease duration at baseline was 1-2 months in 4.4% (n = 67/1510) of patients, 3-5 months in 5.0%, 6-11 months in 8.5%, 1-4 years in 38.3%, and ≥ 5 years in 43.7%, with a median duration of 3.5 years (IQR 1.0-8.5 years, mean ± standard deviations 6.6 ± 8.2 years). A current comorbid disease was present in 76.4% (n = 1,154/1,510) of patients, with a median of 1.0 (IQR 1.0-2.0) comorbid diseases per patient. The most common comorbid diagnoses were M00-M99 Diseases of the musculoskeletal system and connective tissue (14.7%, n = 350/2,378 diagnoses), F00-F99 Mental and behavioural disorders (13.6%), J00-J99 Diseases of the respiratory system (9.4%), I00-I99 Diseases of the circulatory system (8.3%), and E00-E99 Endocrine, nutritional and metabolic diseases (8.1%).

**Table 2:** Most frequent diagnosis groups

**Diagnosis**	**N**	**Age years**	**Recruitment period**	**Criteria**	**Outcome measures (range)**	**Previous analysis**
Depression	135	17-70	1999-2005*	Depressed mood plus ≥2 of 6 defined core symptoms of depression; symptom duration ≥ 6 months; CES-D ≥ 24 points	CES-D (0-60)	[24]
Asthma	90	2-70	1999-2005	Physician’s diagnosis (ICD-10 J45)	Average Asthma Severity (NRS, 0-10)	[28]
Low Back Pain	75	17-75	1999-2005	Low back pain of ≥ 6 week duration. Exclusion: previous back surgery, 11 specific diagnoses	HFAQ (0-100), LBPRS (0-100),	[26]
Anxiety Disorders	64	17-75	1999-2005	Physician’s diagnosis (ICD-10 F40-F42 or F43.1)	Anxiety Severity (NRS, 0-10)	[27]
ADHD Symptoms	61	3-16	2001-2005	Physician’s diagnosis (ICD-10 F90), symptom duration ≥ 6 months	FBB-HKS Total score (0-3)	[29]
Migraine	45	17-75	1999-2005	Criteria of the International Headache Society [50]	Average Migraine Severity (NRS, 0-10)	[30]

*CES-D* Center for Epidemiological Studies Depression Scale, German version, *ICD-10* International Classification of Diseases, Tenth Revision. *Recruitment period for previously published 4-year analysis [24]: 1998-2001. *NRS* Numerical rating scales [32], *HFAQ*, Hanover Functional Ability Questionnaire [51], *LBPRS* Low Back Pain Rating Scale Pain Score [52], *ADHD* Attention Deficit Hyperactivity Disorder, *FBB-HKS* Fremdbeurteilungsbogen für Hyperkinetische Störungen (parents’ questionnaire for ADHD core symptoms) [53,54].

### Therapy

At enrolment 19.4% (n = 293/1,510) of patients fulfilled inclusion criterion 2a (AM-related consultation of ≥ 30 minutes followed by new prescription of AM medication), 44.3% fulfilled inclusion criterion 2b (referral to AM eurythmy/art/massage therapy), and 36.3% fulfilled inclusion criteria 2a and 2b. The duration of the consultation with the AM physician at enrolment was < 30 min in 51.4% (n = 776/1,510) of patients, 30-44 min in 23.6%, 45-59 min in 11.5%, and ≥ 60 min in 13.5% of patients.

Of the 1,217 patients who were referred to AM art, eurythmy or massage therapy, 86.8% (n = 1,065) had the planned AM therapy within the first 24 months, 0.5% did not have AM therapy, and for 12.6% the AM therapy documentation is incomplete. AM therapies used were eurythmy therapy (66.4%, n = 707 of 1,065 patients who had the planned AM therapy), rhythmical massage therapy (10.9%), and art therapy (22.7%, n = 242) with the therapy modalities painting/drawing/clay (54.1%, n = 131 of the 242 patients who had art therapy), speech exercises (33.5%), and music (12.4%). The AM therapy started median 13 (IQR 2-41) days after enrolment. Median therapy duration was 119 days (IQR 84-190 days), median number of therapy sessions was 12 (IQR 10-20). AM medications were used by 61.2% (n = 924/1,510) of patients in months 0-6 and by 71.7% in months 0-24.

### Continuity of physician-patient-relationship

At 48-month follow-up 62.8% of the evaluable patients (n = 575/915) were still being treated by the AM physician who had enrolled them into the study. Reasons for no longer being treated by the AM physician were “positive”: full recovery or improvement (13.1% of all evaluable patients, n =120/915), “negative”: choice of other treatment or dissatisfaction with the physician (12.0%, n = 110), and “neutral”: practical reasons, e. g. patient or physician had moved, physician had stopped practicing or financial reasons (9.3%, n = 85), and other reasons (2.7%, n = 25). The 0-48 month Symptom Score improvement did not differ significantly between patients with or without ongoing treatment by the AM physician (p = 0.234), while in the latter group, patients with “positive” reasons for not being treated had more improvement than patients with “negative” reasons (mean difference 1.96 points, 95%-CI 1.29-2.62 points, p < 0.001) (Table 3).

**Table 3:** Symptom score 0-48 months

**Symptom score (0-10)**	**N**	**0 months**	**48 months**	**0-48 month difference**		
		**Mean (95%-CI)**	**Mean (95%-CI)**	**Mean (95% CI)**		**P-value**
All evaluable patients	908	5.96 (5.84-6.07)	2.75 (2.62-2.88)	3.21	(3.05-3.37)	<0.001
Still being treated by study physician?						
-Yes	571	5.85 (5.70-6.01)	2.72 (2.57-2.88)	3.13	(2.94-3.33)	<0.001
-No	337	6.13 (5.94-6.31)	2.79 (2.55-3.03)	3.34	(3.06-3.61)	<0.001
--No: “Positive” reasons	120	6.09 (5.77-6.41)	1.52 (1.28-1.76)	4.57	(4.17-4.96)	<0.001
--No: “Neutral” reasons	108	6.13 (5.81-6.46)	3.43 (3.00-3.85)	2.71	(2.29-3.12)	<0.001
--No: “Negative” reasons	109	6.16 (5.83-6.49)	3.55 (3.09-4.00)	2.61	(2.07-3.15)	<0.001

Respondents at 48-month follow-up with available baseline score. *CI* Confidence interval. “Positive” reasons: full recovery or improvement. “Neutral” reasons: practical reasons (e. g. patient or physician had moved, physician had stopped practicing or financial reasons) or other reasons. “Negative” reasons: choice of other treatment or dissatisfaction with physician.

### Clinical outcomes at 48-month follow-up

**Figure 1:**
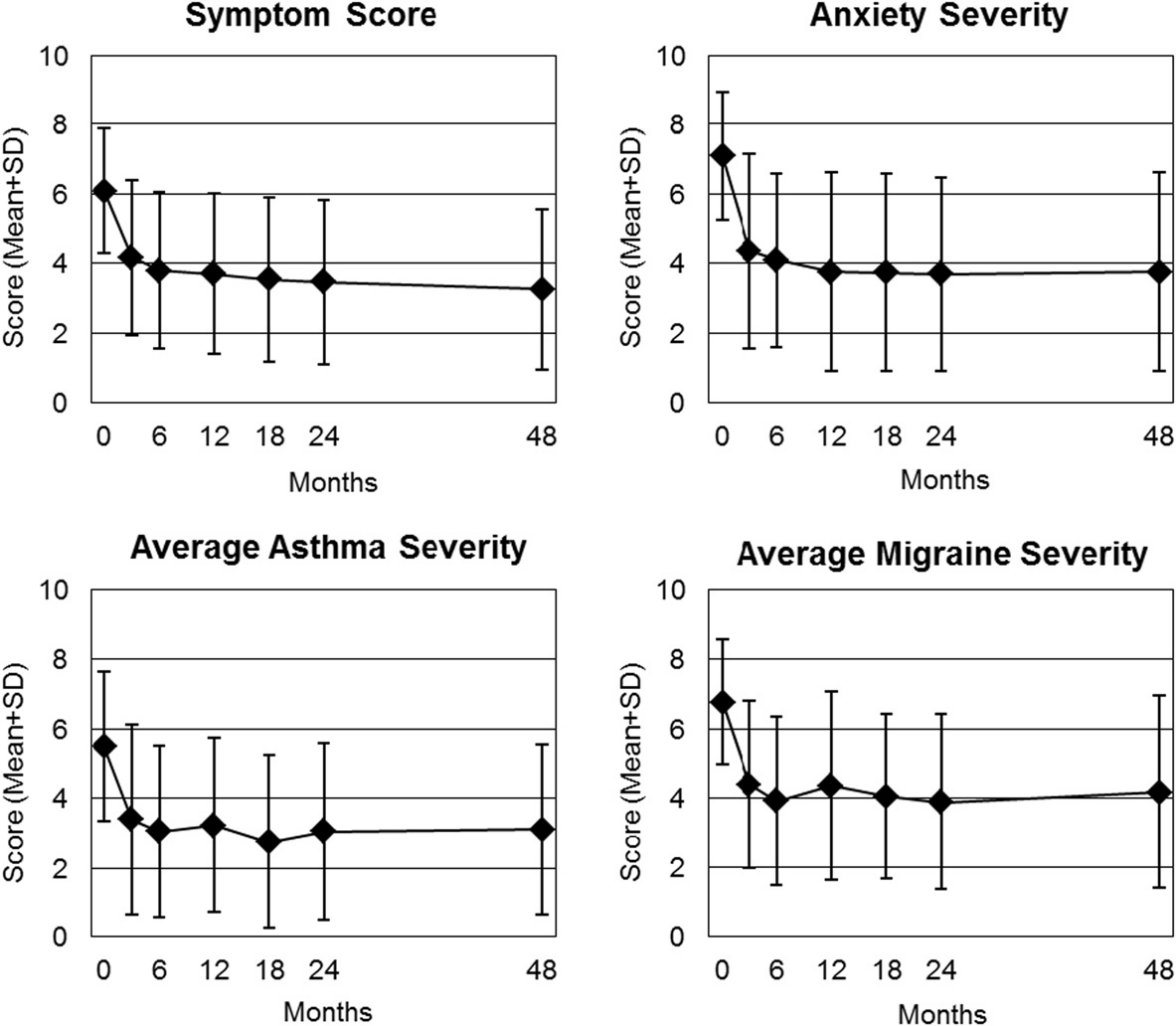
**Clinical outcomes on numerical rating scales (0-10).** Range from 0 (“not present”) to 10 (“worst possible”). Symptom Score: all patients (n = 1,501). Diagnosis groups Anxiety Disorders (n = 61), Asthma (n = 88), and Migraine (n = 44).

**Figure 2:**
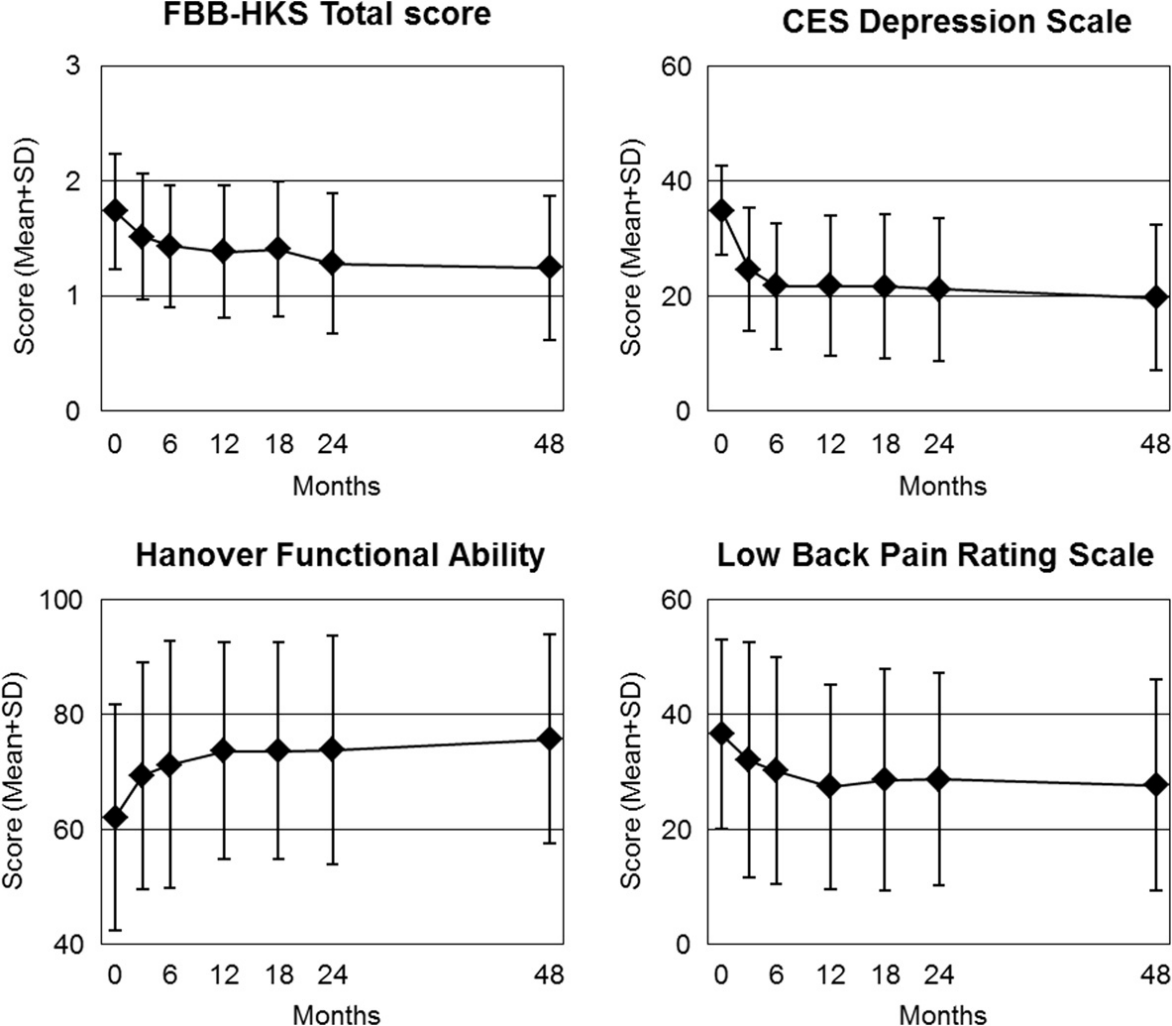
**Clinical outcomes on other scales.** FBB-HKS Total score: Range from 0 (“not present”) to 3 (“very strong intensity”) [53,54] (Attention Deficit Hyperactivity Symptoms group, n = 60). Center for Epidemiological Studies Depression Scale, German version: Range 0-60, higher scores indicate more depressive symptoms [34,35] (Depression group, n = 133). Hanover Functional Ability Questionnaire: Range from 0 (“minimal function”) to 100 (“optimal function”) [51] (Low Back Pain group n = 72). Low Back Pain Rating Scale (LBPRS) Pain Score: Range from 0 (“no pain”) to 100 (“unbearable pain”) [52] (n = 72).

**Figure 3:**
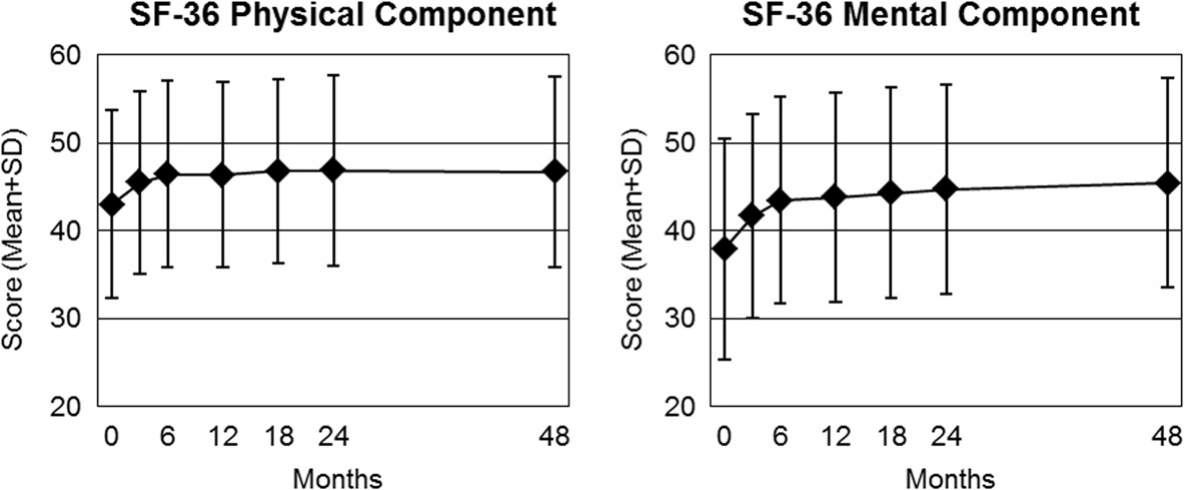
**SF-36 Physical and Mental Component summary measures.** Higher scores indicate better health [33]. Adult patents (n = 1,043).

At 48-month follow-up all clinical outcomes in all analysed groups were significantly improved from baseline (p < 0.001 for all 26 comparisons, Table 4, Figures 1, 2 and 3). For the primary outcome Symptom Score, an improvement of at least 50% of baseline scores was observed in 50.2% (n = 754/1,501) of patients. For symptom assessments (16 comparisons), Standardised Response Mean effect sizes were large for 14 comparisons and medium for two comparisons (CES-D in all adults, Low Back Pain Rating Scale Pain Score in Low Back Pain group). For generic quality of life assessments (SF-36 scores, 10 comparisons), effect sizes were medium for five comparisons and small for five comparisons.

**Table 4:** Clinical outcomes 0-48 months

**Outcome (range)**	**Age years**	**N**	**0 months**	**48 months**	0-48 month difference*		**SRM**
			**Mean (95%-CI)**	**Mean (95%-CI)**	**Mean (95%-CI)**	**P-value**	
**All diagnoses**							
Symptom Score (0-10)							
-All patients	1-75	1501	6.09 (6.00-6.18)	3.26 (3.14-3.37)	2.83 (2.71-2.96)	<0.001	1.13
-Adults	17-75	1067	6.07 (5.96-6.17)	3.41 (3.27-3.54)	2.66 (2.51-2.81)	<0.001	1.09
-Children	1-16	434	6.15 (5.98-6.32)	2.90 (2.68-3.11)	3.25 (3.01-3.50)	<0.001	1.25
-Art therapy	1-75	273	6.19 (6.00-6.38)	3.24 (2.98-3.50)	2.95 (2.68-3.23)	<0.001	1.28
-Eurythmy therapy	1-75	791	6.23 (6.10-6.36)	3.35 (3.18-3.51)	2.88 (2.70-3.06)	<0.001	1.12
-Rhythmical massage Therapy	1-75	146	6.06 (5.78-6.33)	3.56 (3.16-3.96)	2.50 (2.08-2.91)	<0.001	0.98
-Medical therapy	1-75	291	5.63 (5.43-5.84)	2.88 (2.62-3.14)	2.75 (2.47-3.04)	<0.001	1.12
First ranked symptom (0-10)	1-75	1491	6.42 (6.32-6.52)	3.29 (3.29-3.42)	3.13 (2.99-3.28)	<0.001	1.09
CES-D (0-60)	17-75	849	21.83 (21.02-22.64)	15.34 (14.55-16.13)	6.49 (5.72-7.27)	<0.001	0.56
SF-36 Physical Component	17-75	1043	42.98 (42.33-43.68)	46.67 (46.01-47.33)	3.69 (3.11-4.27)	<0.001	0.39
SF-36 Mental Component	17-75	1043	37.90 (37.14-38.66)	45.41 (44.69-46.13)	7.51 (6.75-8.26)	<0.001	0.60
SF-36 scales (0-100)							
-Physical Function	17-75	1069	74.70 (73.28-76.13)	80.30 (78.84-81.76)	5.60 (4.38-6.82)	<0.001	0.28
-Role Physical	17-75	1063	44.75 (42.38-47.12)	66.35 (63.95-68.74)	21.60 (19.03-24.17)	<0.001	0.51
-Role Emotional	17-75	1059	49.32 (46.79-51.86)	70.46 (68.08-72.84)	21.14 (18.44-23.84)	<0.001	0.47
-Social Functioning	17-75	1072	59.17 (57.57-60.76)	74.95 (73.43-76.47)	15.79 (14.12-17.46)	<0.001	0.57
-Mental Health	17-75	1070	53.62 (52.44-54.80)	65.32 (64.12-66.52)	11.70 (10.54-12.86)	<0.001	0.60
-Bodily Pain	17-75	1070	54.19 (52.47-55.91)	67.70 (66.01-69.38)	13.51 (11.84-15.17)	<0.001	0.49
-Vitality	17-75	1070	38.05 (36.93-39.18)	51.72 (50.48-52.97)	13.67 (12.41-14.92)	<0.001	0.65
-General Health	17-75	1061	50.22 (49.04-51.39)	59.12 (57.80-60.45)	8.91 (7.76-10.06)	<0.001	0.47
**Diagnosis groups**							
ADHD: FBB-HKS Total (0-3)	3-16	60	1.74 (1.61-1.87)	1.25 (1.08-1.41)	0.49 (0.34-0.64)	<0.001	0.85
Anxiety Severity (0-10)	17-75	61	7.08 (6.61-7.55)	3.72 (2.99-4.45)	3.36 (2.67-4.06)	<0.001	1.24
Asthma Severity (0-10)	2-70	88	5.50 (5.04-5.96)	3.11 (2.59-3.64)	2.39 (1.86-2.91)	<0.001	0.96
Migraine Severity (0-10)	17-75	44	6.77 (6.23-7.32)	4.18 (3.34-5.02)	2.59 (1.84-3.34)	<0.001	1.05
Depression: CES-D (0-60)	17-70	133	34.93 (33.58-36.27)	19.75 (17.58-21.92)	15.18 (12.94-17.41)	<0.001	1.16
Low back pain: HFAQ (0-100)	17-75	73	62.13 (57.54-66.72)	75.71 (71.48-79.95	13.59 (9.83-17.34)	<0.001	0.84
Low back pain: LBPRS (0-100)	17-75	72	36.61 (32.75-40.46)	27.69 (23.37-32.01)	8.92 (5.11-12.73)	<0.001	0.55

Patients with available baseline values, last value carried forward. *CI* Confidence interval. *Positive differences indicate improvement. *SRM* Standardised Response Mean effect size (minimal: < 0.20, small: 0.20-0.49, medium: 0.50-0.79, large: ≥0.80). *CES-D* Center for Epidemiological Studies Depression Scale, German version [34,35], *ADHD* Attention Deficit Hyperactivity Symptoms, *FBB-HKS Total* Fremdbeurteilungsbogen für Hyperkinetische Störungen (parents’ questionnaire for ADHD core symptoms), Total score [53,54]), *HFAQ* Hanover Functional Ability Questionnaire [51], *LBPRS* Low Back Pain Rating Scale Pain Score [52].

The 0-48 month differences in the present analysis (Table 4) were compared to corresponding long-term pre-post differences of each outcome and subgroup in twelve previously published analyses from the AMOS study [1,20-30]. The compared analyses differed in the following aspects:

•Six previous analyses [1,20-24] comprised patients enrolled 1999-2001, while the present analysis comprised patients enrolled 1999-2005.

•Seven previous analyses [1,25-30] referred to 0-24 month differences, while the present analysis referred to 0-48 month differences.

•In ten previous analyses [1,20-28] the pre-post differences had been calculated as average baseline scores minus average scores at the last follow-up in patients with available scores at baseline and follow-up, respectively, while the present analysis was performed on patients with available scores at baseline after replacement of missing values with the last value carried forward.

Each of the twelve previously published analyses [1,20-30] comprised pre-post differences of one or several clinical outcomes, yielding a total of 24 pre-post differences. These were compared to the 24 corresponding pre-post differences in the present analysis (of 26 pre-post differences in the present analysis [Table 4], two pre-post differences had not been previously published: first ranked symptom, CES-D in all adults). For 21 comparisons the differences between the two analyses were minimal (less than 0.2 standard deviations [SD]) while three comparisons showed small differences indicating less improvement in the present analysis: Symptom Score in rhythmical massage therapy group (0.21 SD difference), Average Asthma Severity (0.29 SD), and Average Anxiety Severity (0.40 SD).

### Other outcomes

#### *Patient therapy outcome rating and satisfaction after 6 and 12 months*

At 6-month follow-up the patients’ ratings of therapy outcome (0 = no help at all, 10 = helped very well, n = 1,275) were mean ± standard deviation 7.23 ± 2.39 points; patient satisfaction with therapy (0 = very dissatisfied, 10 = very satisfied, n = 1,273) was 7.94 ± 2.20 points. Between 6- and 12-month follow-ups therapy outcome ratings did not change significantly, while therapy satisfaction decreased by average 0.31 points (95%-CI 0.18-0.45 points, p < 0.001, n = 1,083).

#### *Safety in months 0-24*

Adverse reactions from AM therapies and medications were infrequent and mostly of mild-to-moderate intensity (Table 5). Adverse drug reactions were reported significantly less frequently from AM medications (4.4% of users, n = 48/1,083) than from non-AM medications (14.8% of users, n = 173/1,167) (p < 0.001, odds ratio for reported reaction from AM vs. non-AM reaction: 0.27, 95%-CI 0.19-0.37).

**Table 5:** Adverse reactions in months 0-24

**Therapy**	**Reported adverse reactions (this analysis)**			
	Users*	**Any reaction**	**Severe intensity**	**Therapy stopped**
	**N**	**N (%)**	**N (%)**	**N (%)**
AM therapies	1065	21 (2.0%)	4 (0.4%)	3 (0.3%)
-Eurythmy	707	13 (1.8%)	2 (0.3%	1 (0.1%)
-Art therapy	242	3 (1.2%)	1 (0.4%)	0 (0.0%)
-Rhythmical massage	116	5 (4.3%)	1 (0.9%)	2 (1.7%)
Non-AM non-medication	No data	26	14	10
Non-AM medication	1167	173 (14.8%)	57 (4.9%)	78 (6.7%)
AM medication	1083	48 (4.4%)	13 (1.2%)	33 (3.0%)
	**Confirmed adverse reactions**[40]			
AM medication	662	20 (3.0%)	2 (0.3%)	10 (1.5%)

*AM* Anthroposophic medicine, *N* patient numbers. *Users of AM therapies: Patients referred to the respective therapy at enrolment who definitely received therapy.

Serious adverse events occurred in 2.3% (n = 34/1,510) of patients. The events were acute hospital admissions (n = 16), deaths (n = 15), new malignancy (n = 1), life-threatening ileus (n = 1), and permanent disability from whiplash injury (n = 1). Death causes in the 15 patients who died were malignant neoplasms (n = 12), AIDS (n = 1), pneumonia (n = 1), and accident or suicide in a patient hospitalised for severe depression (n = 1). None of the serious adverse events were causally related to any medications or therapies.

## Discussion

### Main findings

This is a four-year follow-up analysis of the largest clinical outcome study of AM treatment for chronic disease so far. We studied 1,510 outpatients starting comprehensive AM treatment (physician counselling, art therapy, eurythmy therapy, rhythmical massage therapy and/or AM medications) for mental, musculoskeletal, respiratory or neurological disorders or other chronic conditions. At four-year follow-up, 76% of the evaluable patients were either still being treated by the AM physician who had enrolled them into the study (63% of the patients) or were no longer receiving treatment because they had improved or recovered (13%). Previous analyses of different age, diagnosis and therapy modality groups had shown clinically relevant improvements of symptoms and quality-of-life following AM treatment [1,20-30]. The present follow-up-analysis confirmed these improvements in a larger sample and showed that the improvements were maintained at four-year follow-up. Adverse reactions to AM medications or therapies were infrequent and mostly of mild-to-moderate intensity.

### Strengths and limitations

Strengths of the AMOS study and this analysis include a large sample size, a long follow-up period, the combination of generic and disease-specific outcome measures, the assessment of a broad range of AM therapy modalities, and a high representativeness due to the participation of 47% of eligible AM physicians and 23% of eligible AM therapists in Germany. The participating physicians and therapists resembled eligible but not participating AM physicians and AM therapists with respect to demographic characteristics, and the included patients resembled not included patients regarding baseline characteristics. These features suggest that the study to a high degree mirrors contemporary AM use in German outpatient settings

The main research question of the present analysis concerned the magnitude of long-term improvements. A limitation in this respect is the increasing long-term nonrespondent rate (i. e. proportion of patients not returning the follow-up questionnaire: 17%, 23%, 27%, and 39% after 12, 18, 24, and 48 months, respectively). However, nonrespondents and respondents at 48-month follow-up did not differ regarding baseline characteristics (except nonrespondents had 5% higher symptom intensity at baseline), and in a telephone survey of AMOS patients, the proportion with clinical deterioration at 24-month follow-up was comparable in nonrespondents and respondents [26]. Moreover, a general explanation for late non-responding is the tendency of study subjects to fail to respond to repeated administration of questionnaires. Nonetheless, we cannot exclude that nonrespondents may have showed less improvement than respondents at 48-month follow-up. To suppress such nonrespondent bias, clinical outcomes were analysed after replacing missing values with the last value carried forward. This procedure was based on the assumption of a maximum plausible extent of nonrespondent bias, namely that in nonrespondents at 48-month follow-up, Symptom Score would show an average of zero change from the last available score value, which was documented after average 15.7 months. The assumed average zero change among 48-month nonrespondents can be compared to the observed change among 48-month respondents in the same period, which was an average of 19% improvement. Considering this finding, and with nonrespondent analyses showing no relevant differences between respondents and nonrespondents, it appears unlikely that non-respondents should have shown an average deterioration. Accordingly, the replacement of missing values with the last value carried forward, as performed in this analysis, seems an appropriate conservative measure in regard to nonrespondent bias [55].

This was an exploratory analysis and a total of 19 clinical outcomes were analyzed with a total of 26 pre-post comparisons (Table 4), therefore the issue of multiple hypothesis-testing arises [36]. However, all 26 comparisons showed significant improvements with p-values < 0.001 – a constellation that would not be expected to occur by chance (e. g. a Bonferroni adjustment for 26 tests would have indicated p < 0.002 as the significance level).

### Relevance of study findings, comparison to other studies

In this analysis, improvements following AM treatment persisted for 48 months. This is in keeping with the aims of AM therapy to bring about sustained improvement [6,7,56]. Long-term clinical outcomes of AM therapy for chronic disease has been evaluated in four studies by other researchers [57-60], with follow-up periods of 12 months [57,58], 14 months [60], and 36 months [59], respectively. These studies investigated AM medications [60] or comprehensive AM therapy [57-59] for anxiety in adult cancer patients [57] and asthma in children [58-60] and adults [60], treated in inpatient hospitals [57], outpatient clinics [58-60] or primary care [59]. All studies showed long-term improvement following AM treatment. In accordance with these studies, mostly from specialized settings, this 48-month follow-up analysis from the AMOS study in a predominantly primary care setting showed substantial improvements in symptoms and quality of life for patients with a broader range of chronic indications. Long-term improvements in symptoms and quality of life have also been observed in patients receiving other complementary therapies for similar chronic indications [61-64].

In this study, adverse reactions to AM treatment were infrequent and mostly of mild-to-moderate intensity. This confirms findings from other prospective studies [7,65-68] as well as retrospective surveys [69] and pharmacovigilance databases [70]. Also, the high level of patient satisfaction with AM treatment in this study is in keeping with other studies [67,71-73]. Finally, the present 48-month follow-up analysis showed a high degree of continuity of the physician-patient-relationship in AM settings, a quality which is important to physicians and patients [74].

To sum up: This study confirms the findings of similar research conducted elsewhere [7,57-73] and further strengthens the importance of a role for complementary therapies in chronic disease and the evaluation of complementary treatment in long-term naturalistic outcome studies [75,76].

## Conclusions

This 48-month follow-up analysis confirmed previous analyses from the AMOS study. Outpatients receiving comprehensive AM treatment for chronic indications had sustained, clinically relevant improvements of symptoms and quality of life. Adverse reactions to AM treatment were infrequent and mostly of mild-to-moderate intensity; patient satisfaction was high.

## Abbreviations

± and SD: Standard deviation; ADHD: Attention deficit hyperactivity disorder; AM: Anthroposophic medicine; AMOS: Anthroposophic medicine outcomes study; CES-D: Center for epidemiological studies depression scale, German version; CI: Confidence interval; ICD-10: International classification of diseases, Tenth Edition; IQR: Interquartile range; NRS: Numerical rating scale.

## Competing interests

All authors declare that they have no competing interests.

## Authors’ contributions

HJH, HK and GSK contributed to study design. HJH, AG, and HK contributed to data collection. HJH, RZ, and HK wrote the analysis plan, HJH and AG analysed data. HJH was principal author of the paper, had full access to all data, and is guarantor. All authors contributed to manuscript drafting and revision and approved the final manuscript.
